# Supplementary material for: Analysis of contemporary value and influence of intangible cultural heritage based on online review mining
Source: PLoS One. 2024 Dec 19;19(12):e0315805. doi: 10.1371/journal.pone.0315805 (PMC11658534; doi:10.1371/journal.pone.0315805)
Supplement: Attachment — Submitted filename: Response to Reviewers.docx [file pone.0315805.s001.docx]

**Response to Reviewers**

**Title of the Manuscript:** Analysis of Contemporary Value and Influence of Intangible Cultural Heritage Based on Online Review Mining

**Manuscript ID:** PONE-D-24-01376

Dear Dr. Hsu,

We appreciate the time and effort that the reviewers have dedicated to critiquing our manuscript. We have carefully considered each comment and have made several revisions to the manuscript accordingly. Below, we provide a detailed response to each of the comments raised by the reviewers.

**To academic editor’ comments:**

**Comment 1:**

Please ensure that your manuscript meets PLOS ONE's style requirements, including those for file naming.

**Response:**

Thank you for your notice. We have updated the manuscript to meet the requirements of PLOS ONE’s style.

**Comment 2:**

Please note that PLOS ONE has specific guidelines on code sharing for submissions in which author-generated code underpins the findings in the manuscript. In these cases, all author-generated code must be made available without restrictions upon publication of the work.

**Response:**

Thank you for your advice. We have uploaded and shared the code and it can be accessed by DOI: 10.5281/zenodo.11004055

**Comment 3:**

Please state what role the funders took in the study. If the funders had no role, please state: "The funders had no role in study design, data collection and analysis, decision to publish, or preparation of the manuscript." If this statement is not correct you must amend it as needed. Please include this amended Role of Funder statement in your cover letter; we will change the online submission form on your behalf.

**Response:**

Thank you for your notice. We have updated the founder’s role and contribution in the cover letter. In case it was not seen, we also state it separately here.

1.the National Natural Science Foundation of China (No. 72104219): Study design, data collection and analysis

2.the National Natural Science Foundation of China (No. 62207016): data collection and analysis

3.the MOE Project of Humanities and Social Sciences (No. 21YJC870013): preparation of the manuscript

4.Major Humanities and Social Sciences Research Projects in Zhejiang Higher Education Institutions (No. 2023QN129): preparation of the manuscript, decision to publish.

**Comment 4:**

In the online submission form, you indicated that your data will be submitted to a repository upon acceptance. We strongly recommend all authors deposit their data before acceptance, as the process can be lengthy and hold up publication timelines. Please note that, though access restrictions are acceptable now, your entire minimal dataset will need to be made freely accessible if your manuscript is accepted for publication. This policy applies to all data except where public deposition would breach compliance with the protocol approved by your research ethics board. If you are unable to adhere to our open data policy, please kindly revise your statement to explain your reasoning and we will seek the editor's input on an exemption.

**Response:**

Thank you for your advice. We have uploaded and shared the data and it can be accessed by DOI: 10.5281/zenodo.11004089.

**Comment 5:**

We note that Figures 4 and 5 in your submission contain copyrighted images. All PLOS content is published under the Creative Commons Attribution License (CC BY 4.0), which means that the manuscript, images, and Supporting Information files will be freely available online, and any third party is permitted to access, download, copy, distribute, and use these materials in any way, even commercially, with proper attribution.

**Response:**

Thank you for your notice. This two figures Figure 4 and 5 have been generated and edited through a public data visualization platform: <https://wordart.com/>. The data used are all our own data and have been set to public access. So, there is no copyright issue to be concerned.

**To reviewers' comments:**

**Reviewer #1:**

**Comment:**

Accept the Article entitled "Analysis of Contemporary Value and Influence of Intangible Cultural Heritage Based on Online Review Mining". This article addressing Influence of Intangible Cultural Heritage Based on Online Review Mining.

**Response:**

Thank you for your recognition of our research.

**Reviewer #2:**

**Comment:**

Valuable paper for this era. The examination of ICH's current significance and impact reveals its varied function in fostering innovation, social cohesiveness, cultural diversity, and sustainable development. Communities may take advantage of ICH's potential to improve lives and build a more inclusive and sustainable future by realising its significance and supporting its promotion and preservation.

**Response:**

Thank you for your recognition of our research.

**Reviewer #3:**

**Comment:**

The authors have used machine learning techniques for ICH (Intangible Cultural Heritage) value perception classification task. The manuscript lacks substantial data and methodology, such as details on neural network architectures (e.g., BERT, Bi-GRU RNN).

**Response:**

Thank you for your precious advice. We have modified and improved the BERT-based word vector technology and the neural network structure involved in the classification model, especially the core structure of the GRU unit.

We believe the revisions we have made address the reviewers’ concerns comprehensively and enhance the manuscript significantly. Attached, you will find the revised manuscript along with a marked-up copy highlighting the changes made.

We thank you for the opportunity to revise our manuscript and hope that the changes meet your and the reviewers' satisfaction.

Best regards,

Dr. Chao Ma

College of Economic and Management, Zhejiang Normal University

Machao456@hotmail.com
