# Supplementary material for: Analysis of contemporary value and influence of intangible cultural heritage based on online review mining
Source: PLoS One. 2024 Dec 19;19(12):e0315805. doi: 10.1371/journal.pone.0315805 (PMC11658534; doi:10.1371/journal.pone.0315805)
Supplement: Attachment — Submitted filename: Response to Reviewers.docx [file pone.0315805.s002.docx]

**Response to Reviewers**

**Title of the Manuscript:** Analysis of Contemporary Value and Influence of Intangible Cultural Heritage Based on Online Review Mining

**Manuscript ID:** PONE-D-24-01376R1

Dear Dr. Riccardo Ortale,

We appreciate the time and effort that the reviewers have dedicated to critiquing our manuscript. We have carefully considered each comment and have made several revisions to the manuscript accordingly. Below, we provide a detailed response to each of the comments raised by the reviewers.

**To reviewers' comments:**

**Reviewer #4:**

**Comment:**

1. The comparative methods are not SOTA. Please add some SOTA classification methods to compare.

2. Four figures of word cloud may not depict your results. add some Tables to show your high-frequency words.

**Response:**

Thank you for your precious advice.

1. According to your first comment, we added two classification methods experiment, the BERT pre-train model and the TEXT-RCNN model are selected as the SOTA methods for comparison.

2. According to your second comment, we added tables to present high-frequency keywords from each category.

Thank you again for your careful review of our manuscript, and we hope you will be satisfied with the revision.

**Reviewer #5:**

**Comment:**

The proposed BiGRU-Attention model, enhanced with BERT word embeddings, aims to achieve effective text contemporary value perception recognition based on user-generated content.

All comments have been answered.

**Response:**

Thank you for your recognition of our research.

**Reviewer #6:**

**Comment:**

This study analyzed real online reviews from the Bilibili website. The design model classified the contemporary values of intangible cultural heritage. The study is instructive, but requires minor revisions.

1. Ensure the quality of article formatting and reference citation.

2. A thorough review and revision for clarity, grammar, and overall language quality are necessary.

3. The discussion of the research results is insufficient. Explain how the results contribute to the existing body of knowledge and highlight their significance.

4. Describe how the findings can be applied in real-world scenarios and their potential impact on the field.

**Response:**

Thank you for your precious advice.

1. According to your first and second comments, we have reviewed and revised our text and format. We have carefully checked the format requirements of the journal and improved them. We hope that you will be satisfied.

2. According to your third and fourth comments, we have added some discussion about the implications of this study for the existing knowledge system and the role it can play in practical environments in the conclusion of the article. The additional discussion has been marked in red, and we hope you will be satisfied.

Thank you again for your careful review of our manuscript.

We believe the revisions we have made address the reviewers’ concerns comprehensively and enhance the manuscript significantly. Attached, you will find the revised manuscript along with a marked-up copy highlighting the changes made.

We thank you for the opportunity to revise our manuscript and hope that the changes meet your and the reviewers' satisfaction.

Best regards,

Dr. Chao Ma

College of Economic and Management, Zhejiang Normal University

Machao456@hotmail.com
