# Supplementary material for: Analysis of contemporary value and influence of intangible cultural heritage based on online review mining
Source: PLoS One. 2024 Dec 19;19(12):e0315805. doi: 10.1371/journal.pone.0315805 (PMC11658534; doi:10.1371/journal.pone.0315805)
Supplement: Attachment — Submitted filename: Response to Reviewers.docx [file pone.0315805.s003.docx]

**Response to Reviewers**

**Title of the Manuscript:** Analysis of Contemporary Value and Influence of Intangible Cultural Heritage Based on Online Review Mining

**Manuscript ID:** PONE-D-24-01376R2

Dear Dr. Riccardo Ortale,

We appreciate the time and effort that the reviewers have dedicated to critiquing our manuscript. We have carefully considered each comment and have made several revisions to the manuscript accordingly. Below, we provide a detailed response to each of the comments raised by the reviewers.

**To reviewers' comments:**

**Reviewer #6:**

**Comment:**

The authors have basically solved the existing problems and has some suggestions. Why did the authors mark cultural value, social value, and economic value? What is the basis of this classification?

**Response:**

Thank you for your careful review. Based on your valuable suggestions, we have added a discussion on the source of classification, and we have added a theoretical basis of Hofstadter's cultural value and related references. This is the basis for the classification of the perception of intangible cultural heritage value.

We believe the revisions we have made address the reviewers’ concerns comprehensively and enhance the manuscript significantly. Attached, you will find the revised manuscript along with a marked-up copy highlighting the changes made.

We thank you for the opportunity to revise our manuscript and hope that the changes meet your and the reviewers' satisfaction.

Best regards,

Dr. Chao Ma

College of Economic and Management, Zhejiang Normal University

Machao456@hotmail.com
