# Supplementary material for: Analysis of contemporary value and influence of intangible cultural heritage based on online review mining
Source: PLoS One. 2024 Dec 19;19(12):e0315805. doi: 10.1371/journal.pone.0315805 (PMC11658534; doi:10.1371/journal.pone.0315805)
Supplement: Attachment — Submitted filename: Response to Reviewers.docx [file pone.0315805.s004.docx]

**Response to Reviewers**

**Title of the Manuscript:** Analysis of Contemporary Value and Influence of Intangible Cultural Heritage Based on Online Review Mining

**Manuscript ID:** PONE-D-24-01376R3

Dear Dr. Riccardo Ortale,

We appreciate the time and effort that the reviewers have dedicated to critiquing our manuscript. We have carefully considered each comment and have made several revisions to the manuscript accordingly. Below, we provide a detailed response to each of the comments raised by the reviewers.

**To reviewers' comments:**

**Reviewer #7:**

**General Overview**

**Comment:** The manuscript presents an innovative approach to analyzing how intangible cultural heritage (ICH) is perceived and valued through the analysis of online reviews. It employs data mining methods and deep learning models, such as BiGRU with attention, to identify the cultural, social, and economic dimensions of ICH value.

**Response:** Thank you for your positive feedback on the methodology and the innovative approach of the study. We are pleased that you found the analysis of online reviews and the use of advanced models like BiGRU with attention appropriate for identifying the cultural, social, and economic dimensions of ICH value.

**Strengths：**

**Advanced Methodology**

**Comment:** The use of the BiGRU-Attention model with BERT-based embeddings is relevant and well-justified, providing an in-depth semantic analysis.

**Response:** We are grateful for your acknowledgment of the BiGRU-Attention model and the BERT-based embeddings. We believe that these advanced methods significantly enhance semantic analysis, and we have made minor clarifications in the revised manuscript to ensure the methodology’s application is fully explained.

**Multidimensional Approach**

**Comment:** The segmentation of value perceptions into cultural, social, and economic adds depth to the analysis and allows for a comprehensive understanding of ICH in the contemporary context.

**Response:** Thank you for your positive comments on the multidimensional approach. We have added more detail to the explanation of how we define and categorize cultural, social, and economic dimensions in the revised manuscript to enhance clarity and understanding.

**Areas for Improvement：**

**Expansion of the Theoretical Framework**

**Comment:** While the manuscript provides a solid theoretical context, including additional studies on the interaction of ICH with legal, economic, and cultural frameworks would strengthen the argument.

**Response:** We appreciate your suggestion to expand the theoretical framework. In response, we have included several additional studies that explore the interaction of ICH with legal, economic, and cultural frameworks. These additions further strengthen the theoretical context and provide a more comprehensive view of ICH in the digital age.

**Citation Recommendations**

**Comment:**

Heredia-Carroza, J., Palma Martos, L. A., & Aguado, L. F. (2023). This article addresses the challenge of protecting ICH through copyright and examines how these regulations impact the perception and value of heritage.

Heredia-Carroza, J., Palma, L., de Sancha-Navarro, J., & Chavarría-Ortiz, C. (2023). This study analyzes the consumption habits of traditional music, offering relevant insights into the economic factors influencing the acquisition and perceived value of ICH.

Heredia-Carroza, J., Saraiva, H., & Chavarría-Ortiz, C. (2021). This paper presents an economic approach to measuring the value of flamenco performers, providing a quantitative perspective that could enrich the discussion on the economic impacts of ICH.

Han, J. H., & Bae, S. Y. (2022). This article offers an interesting perspective on how authenticity and nostalgia influence tourists' behavior in sharing ICH experiences on social media.

**Response:** We greatly appreciate the citation recommendations you provided. We have carefully reviewed these articles and integrated them into the revised manuscript. These references have been incorporated in relevant sections to provide a more interdisciplinary perspective, as you suggested.

**Deeper Discussion**

**Comment:** The discussion section could benefit from more comparisons with previous studies. Integrating the recommended references would help better position the findings within the current research context and demonstrate how online review analysis contributes to understanding ICH from an interdisciplinary approach.

**Response:** Thank you for this insightful suggestion. We have expanded the discussion section to include comparisons with relevant studies, particularly focusing on how our findings align with or differ from existing research in ICH, cultural economics, and digital heritage studies. The newly integrated references enrich our discussion and situate our work within a broader scholarly context, particularly in relation to legal, economic, and cultural perspectives on ICH.

**Practical Implications and Recommendations**

**Comment:** Including a section that discusses the practical implications of the findings for cultural policymakers, heritage managers, and academics would be valuable. This section could highlight how the results could inform the preservation of ICH and its integration into the digital environment.

**Response:** We agree that discussing the practical implications of our findings is essential. We have added a new section on Practical Implications and Recommendations, where we highlight how our study can inform cultural policymakers, heritage managers, and academics. We discuss how digital platforms can be leveraged to preserve and promote ICH, the importance of balancing copyright with public access, and how the findings can guide heritage management strategies in the digital age. We believe this addition significantly enhances the manuscript’s relevance for practitioners.

**Limitations and Future Research**

**Comment:** Although the manuscript briefly touches on limitations, expanding this section to include specific aspects related to potential biases in online reviews and how they might influence the representation of ICH would be beneficial. Future research directions could explore the application of other data analysis techniques to validate and extend the results.

**Response:** Thank you for your valuable feedback on the limitations section. We have expanded this section to discuss potential biases in online reviews, such as demographic factors, emotional biases, and platform-specific algorithms, which may influence the representation of ICH. Additionally, we have suggested future research directions, including the application of advanced techniques such as sentiment analysis and machine learning to validate our findings and address the limitations of our current approach. These additions provide a clearer direction for future work in this area.

**Conclusion**

**Comment:** The manuscript is a valuable contribution to the study of contemporary perceptions of ICH through digital data analysis. Citing the recommended studies would strengthen the theoretical framework and enrich the discussion, aligning the work with current debates on the legal, cultural, and economic aspects of intangible heritage.

**Response:** We sincerely appreciate your positive assessment of our manuscript. As mentioned, we have incorporated the recommended references to strengthen the theoretical framework and deepen the interdisciplinary discussion on ICH. These additions have helped position our work more clearly within the current academic debates on ICH's legal, economic, and cultural dimensions.

We believe the revisions we have made address the reviewers’ concerns comprehensively and enhance the manuscript significantly. Attached, you will find the revised manuscript along with a marked-up copy highlighting the changes made.

We thank you for the opportunity to revise our manuscript and hope that the changes meet your and the reviewers' satisfaction.

Best regards,

Dr. Chao Ma

College of Economic and Management, Zhejiang Normal University

machao456@hotmail.com
